# Comparing 24 h Urine and Spot Urine Calcium Measurements in Clinical Routine: Accuracy and Limitations

**DOI:** 10.3390/jcm15103901

**Published:** 2026-05-19

**Authors:** Antonia Mondorf, Rejane Golbach, Ludwig Hofbauer, Christine Koch, Christiana Graf, Anna Katharina Flügel, Nora Ackermann, Christian Vorländer, Matthias Pirlich, Christoph Terkamp, Katharina Holzer, Ulrich Mondorf, Alexander Mann, Jörg Bojunga

**Affiliations:** 1 Department of Internal Medicine 1, Goethe University Hospital Frankfurt, Goethe-University, 60590 Frankfurt am Main, Germany; 2 Institute of Biostatistics and Mathematical Modelling, University Hospital, Goethe University, 60590 Frankfurt am Main, Germany; 3 Medicover Berlin-Mitte MVZ, 12159 Berlin, Germany; 4 Department of Endocrine Surgery, Bürgerhospital Frankfurt, 60590 Frankfurt am Main, Germany; 5 Praxis Kaisereiche Berlin, 12159 Berlin, Germany; 6 Department of Gastroenterology, Hepatology, Infectious Diseases and Endocrinology, Hannover Medical School, 30625 Hannover, Germany; 7 Viszeral-, Thorax und Gefäßchirurgie, Section of Endocrine Surgery, Marburg University and Clinics, 35043 Marburg, Germany; 8 Praxis PD Dr. Med. Ulrich Mondorf, 60590 Frankfurt am Main, Germany; 9 Endokrinologikum Frankfurt, 60590 Frankfurt am Main, Germany

**Keywords:** hypercalciuria, urine specimen collection, calcium metabolism, parathyroid disease, nephrolithiasis, 24 h urine collection, spot urine, calcium-to-creatinine ratio

## Abstract

**Background/Objectives**: Urinary calcium excretion is a key parameter in assessing mineral metabolism and diagnosing conditions such as nephrolithiasis, osteoporosis, and hyperparathyroidism. The 24 h urine collection is the gold standard for evaluating calcium excretion, but it is often impractical due to patient non-compliance and logistical challenges. As an alternative, the calcium-to-creatinine ratio (CCR) in spot urine has been proposed, although its reliability remains debated. This study aims to systematically compare the calcium levels in spot urine samples with those obtained from 24 h urine collections to assess their agreement and clinical applicability. **Methods**: This retrospective, multi-center study analyzed data from 201 patients who provided both 24 h and spot urine samples during routine diagnostic work-up between 1 January 2019 and 31 December 2024. Calcium excretion was normalized using the calcium-to-creatinine ratio (CCR). The agreement between the two methods was assessed using Bland–Altman analysis, Pearson and Spearman correlation coefficients, and receiver operating characteristic (ROC) curve analysis. **Results:** Hypercalciuria, defined as ≥6.25 mmol/24 h in women and ≥7.5 mmol/24 h in men, was detected in 52.7% of cases based on 24 h urine. ROC analysis showed that spot urine CCR had moderate diagnostic accuracy (AUC = 0.76). The optimal cut-off for predicting hypercalciuria was 4.4 mmol/g (sensitivity 70.8%, specificity 72.4%). Overall agreement between spot urine CCR and 24 h urine CCR was moderate, with a Bland–Altman geometric mean ratio of 1.06 and multiplicative limits of agreement of 0.59 to 1.91. A low spot urine CCR below 2 mmol/g showed high sensitivity but low specificity and had a negative predictive value of 82%. **Conclusions**: Spot urine CCR cannot replace 24 h urine collection for accurately assessing urinary calcium excretion, but very low values may have limited utility as an initial rule-out tool in selected patients. Very low spot urine CCR values may help rule out hypercalciuria in a limited subgroup of patients and may therefore support triage decisions in selected clinical situations. Further prospective studies are needed to validate these findings.

## 1. Introduction

Calcium excretion in urine is a crucial parameter in the assessment of mineral metabolism and the diagnosis of disorders such as nephrolithiasis, osteoporosis, and hyperparathyroidism [1,2,3].

The gold standard for measuring calcium excretion is 24 h urine collection. A urinary calcium excretion ≥ 6.25 mmol/24 h in women, ≥7.5 mmol/24 h in men, or ≥0.1 mmol/kg/24 h is considered hypercalciuria [2,4,5,6].

Urinary calcium excretion is influenced by various physiological and pathological factors. A key hormonal regulator of urinary calcium excretion is parathyroid hormone (PTH). PTH increases renal tubular calcium reabsorption, primarily in the distal convoluted tubule, thereby reducing urinary calcium excretion. In states involving elevated PTH levels, such as primary hyperparathyroidism, this regulatory mechanism may become dysregulated, contributing to altered calcium homeostasis and, paradoxically, sometimes increased urinary calcium due to overall calcium overload [7,8]. Conversely, low PTH levels, as seen in hypoparathyroidism, are associated with increased urinary calcium loss. The dietary intake of high amounts of calcium, as well as high sodium and protein intake, increases calcium excretion [9,10,11]. The urinary calcium concentration also depends on the hydration status and the body’s acid–base balance and can be influenced by certain medications, especially diuretics; thiazide diuretics reduce urinary calcium loss, whereas loop diuretics such as furosemide increase it [12]. Renal function is another key determinant, as impaired kidney function affects calcium reabsorption and excretion. To compensate for the dilution effect, it is useful to determine the calcium–creatinine ratio (CCR) or the calcium–creatinine clearance rate (CCCR) [13,14]. The cut-off level for hypercalciuria is 5 mmol calcium/1 g creatinine or above 2% [6,9]. CCCR can also be crucial for the differential diagnosis of familial hypocalciuric hypercalcemia (FHH) or other genetic diseases in comparison to primary hyperparathyroidism [15].

Given the numerous influencing factors, 24 h urine collection has traditionally been regarded as the gold standard for assessing urinary calcium excretion [2]. However, this method is often impractical due to patient non-compliance, collection errors, and the logistical challenges associated with sample handling [16,17]. As an alternative, spot urine calcium measurements, particularly when adjusted for creatinine concentration, have been proposed as more convenient and feasible for clinical practice [13,14].

Despite the advantages of spot urine, the reliability of spot urine calcium as a surrogate for 24 h urinary calcium excretion remains contentious. Previous studies have reported varying degrees of correlation between these methods, with potential influences from factors such as dietary intake, circadian rhythm, and renal handling of calcium [18]. Most previous studies have focused on selected populations, such as children, patients with urolithiasis, or postmenopausal women. Data from heterogeneous adult cohorts reflecting real-world endocrine, nephrological, and surgical practice remain limited.

The aim of this study is to systematically compare calcium levels in spot urine samples with those obtained from 24 h urine collections to determine their agreement and clinical applicability. In addition, we evaluated whether spot urine CCR could identify patients at low probability of hypercalciuria and thereby serve as a limited screening or rule-out tool rather than a replacement for 24 h urine collection.

## 2. Materials and Methods

### 2.1. Study Population

This retrospective, multi-center analysis included 201 patients from 7 medical institutions (4 outpatient endocrinological clinics, 1 outpatient nephrological clinic, and 2 inpatient clinics for endocrine surgery) who provided both a 24 h urine sample and a spot urine sample as part of routine medical assessment between 1 January 2019 and 31 December 2024. The participating institutions were: Department of Internal Medicine 1, Goethe University Hospital Frankfurt (inpatient); Medicover Berlin-Mitte, MVZ, Berlin (outpatient endocrinology); Praxis Kaisereiche Berlin, Berlin (outpatient endocrinology); Endokrinologikum Frankfurt, Frankfurt am Main (outpatient endocrinology); Department of Endocrine Surgery, Bürgerhospital Frankfurt, Frankfurt am Main (inpatient); Department of Gastroenterology, Hepatology, Infectious Diseases and Endocrinology, Hannover Medical School, Hannover; and Viszeral-, Thorax-und Gefäßchirurgie, Section of Endocrine Surgery, Marburg University and Clinics, Marburg (inpatient endocrine surgery).

Patients were included if they were aged ≥18 years, had both a 24 h urine sample and a spot urine sample collected, and had complete laboratory data for calcium and creatinine measurements for both samples as well as sufficient clinical and laboratory information to classify hypercalciuria according to sex-specific 24 h urine thresholds. Spot urine and 24 h urine samples were obtained during the same diagnostic episode, with an allowable interval of no more than 7 days.

Patients with missing paired calcium or creatinine measurements were excluded from the primary analysis. For sensitivity analyses, patients were excluded if there was evidence suggesting incomplete or unreliable 24 h urine collection, including 24 h urine volume < 500 mL, 24 h urinary creatinine excretion < 0.01 g/kg body weight/day, estimated glomerular filtration rate < 60 mL/min/1.73 m^2^, or missing data required for these assessments.

Patients received standardized written and verbal instructions for 24 h urine collection according to institutional protocols. Patients were instructed to discard the first morning void, record the start time, and collect all subsequent urine for 24 h including the first morning urine the following day. Spot urine samples were collected as a single midstream urine sample as part of the medical presentation regardless of the time of day and without special diet or drinking instructions.

Patient data were collected anonymously.

### 2.2. Laboratory Tests

The laboratory diagnostics were performed according to the quality standards required in Germany. All procedures adhered to the guidelines outlined in ISO 15189 [19], ensuring that the laboratory met the necessary requirements for quality and competence. Additionally, the laboratory is accredited by the Deutsche Akkreditierungsstelle (DAkkS) based on ISO 15189, confirming compliance with international standards for medical laboratories. Quality control measures were rigorously followed, in line with the requirements of the German Society for Clinical Chemistry and Laboratory Medicine (DGKL), as well as the recommendations provided by the German Medical Association (BÄK). CCR was calculated as urinary calcium divided by urinary creatinine and is expressed as mmol calcium/g creatinine. CCCR was calculated as calcium–creatinine clearance ratio and is expressed as percent. Hypercalciuria was defined as 24 h urinary calcium excretion ≥ 6.25 mmol/24 h (≥250 mg/24 h) in females and ≥7.5 mmol/24 h (≥300 mg/24 h) in males, based on established clinical guidelines [2,3].

### 2.3. Outcomes of This Study

The primary outcome of this study was the agreement between spot urine calcium-to-creatinine ratio (CCR) and 24 h urinary calcium excretion, assessed using Bland–Altman analysis and correlation coefficients. The secondary outcomes were: (1) the diagnostic accuracy of spot urine CCR for identifying hypercalciuria as defined by 24 h urine collection, evaluated by ROC analysis; (2) the performance of a low spot urine CCR threshold as a rule-out strategy for hypercalciuria; and (3) the identification of clinical and laboratory variables associated with discrepancies between the measurement methods, assessed by linear regression analysis.

### 2.4. Statistical Analysis

All data were analyzed using Microsoft Excel, version 2206 (Microsoft, Redmond, WA, USA) and SPSS statistical software, version 30.0.0 (IBM SPSS Statistics, Armonk, NY, USA).

Categorical data are presented as frequencies and percentages. Continuous data are presented as median with interquartile range (Q25–Q75), as variables were not normally distributed.

To assess agreement between spot urine and 24 h urine collection methods, Bland–Altman analysis was performed. Values were log-transformed before calculating the mean difference and limits of agreement (LoA) to meet the assumptions of the analysis. The mean difference and LoA were back-transformed by exponentiation for interpretation on the original scale. The exponentiated mean difference represents the geometric mean ratio between the two methods. The limits of agreement were obtained by exponentiating the upper and lower log-transformed LoA values, which therefore represent the multiplicative range of agreement.

Additionally, an intraclass correlation coefficient (ICC) was calculated using logarithmically transformed values to further assess the consistency between the measurement methods. Pearson’s correlation coefficient was calculated using logarithmically transformed values to evaluate the linear relationship between the methods, while Spearman’s rank correlation coefficient was calculated using non-logarithmized values to assess monotonic relationships. The correlation coefficients provided insight into the degree of association between the spontaneous urine and 24 h urine measurements.

Receiver operating characteristic (ROC) curves were constructed to assess the diagnostic accuracy of spot urine calcium-to-creatinine ratio in identifying hypercalciuria as defined by 24 h urine collection. The area under the curve (AUC) was calculated, and optimal cut-off values were determined. The threshold values for defining abnormal calcium excretion were set at ≥6.25 mmol/24 h in females, ≥7.5 mmol/24 h in males, based on established clinical guidelines [2,3]. Sensitivity, specificity, positive predictive value (PPV), and negative predictive value (NPV) were calculated for different cut-off points.

Linear regression with stepwise backward elimination was performed to analyze factors influencing differences between measurement methods. Variables that did not contribute significantly to the model were sequentially removed to identify the most relevant predictors. Because of the retrospective design, heterogeneity of the cohort, and exploratory nature of the model selection, the regression findings were interpreted cautiously. Variables with *p*-values >0.05 were considered hypothesis-generating rather than statistically significant associations.

All statistical tests were two-sided, and the significance level was set at α = 0.05.

## 3. Results

For the present analysis, data from 201 patients who received a calcium analysis in both 24 h urine and spot urine as part of their medical consultations between 1 January 2019 and 31 December 2024 were analyzed retrospectively. Baseline characteristics are shown in Table 1. Median age of patients was 62 years, with a total of 158 (78.6%) female patients.

The main indications for testing were primary hyperparathyroidism in 133 (66.2%), chronic kidney disease in 30 (14.9%), kidney stones in 29 (14.4%) and osteoporosis in 60 (29.9%) of patients. A total of 17 (8.5%) patients with hypoparathyroidism were included. Average calcium concentration in 24 h urine was 5.7 mmol/d. Hypercalciuria, with a reference range of at >6.25 mmol/24 h in females and >7.5 mmol/24 h in males according to international guidelines [2], was present in 106 (52.7%) of the 201 24 h urine measurements. A total of 32 (15.9%) patients received diuretics (loop diuretics n = 19 (9.5%), thiazide diuretics n = 10 (5.0%)), 108 (53.7%) received vitamin D derivatives and 13 (7.1%) received bisphosphonates.

**Figure A1:**
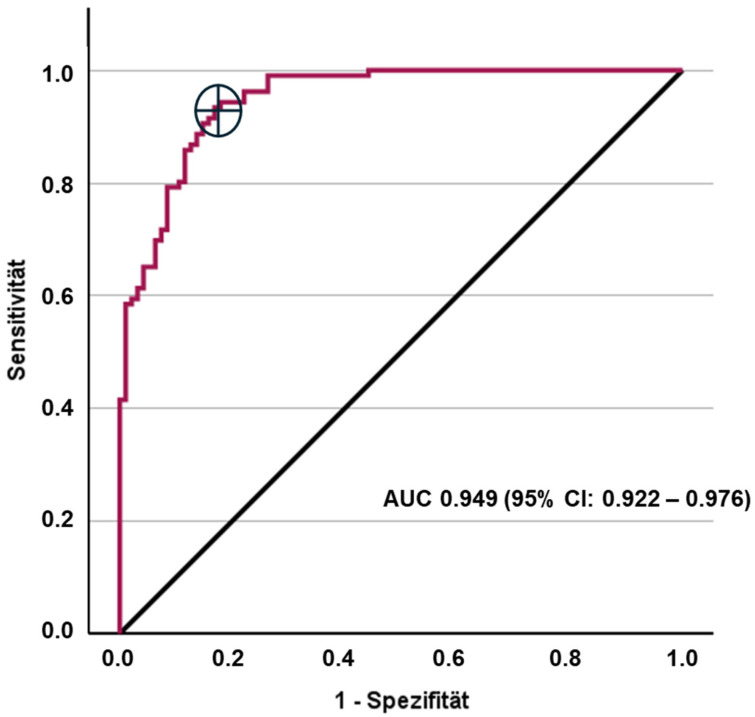
ROC curve, optimal cut-off value, sensitivity, specificity and AUC for 24 h urine CCR to predict hypercalciuria.

The calcium measurements in both the spot and 24 h urine samples were normalized by calculating the calcium-to-creatinine ratio (CCR) to minimize potential variability due to impaired kidney function. Although CCR may be used to normalize urinary calcium excretion for creatinine concentration, international guidelines continue to define hypercalciuria based on total calcium excretion in 24 h urine as the gold standard. Receiver operating characteristic (ROC) analysis in our study population demonstrated a high discriminative ability, with an area under the curve (AUC) of 0.95 (*p* < 0.001). The optimal cut-off value was determined to be 4.8 mmol/g, yielding a sensitivity of 93.4% and a specificity of 83.2%, thereby supporting the previously suggested threshold of 5 mmol/g for 24 h CCR (Appendix A Figure A1). Consequently, CCR in 24 h urine was considered a surrogate measure of total calcium excretion in 24 h urine, demonstrating strong discriminatory performance relative to total calcium excretion.

**Figure 1:**
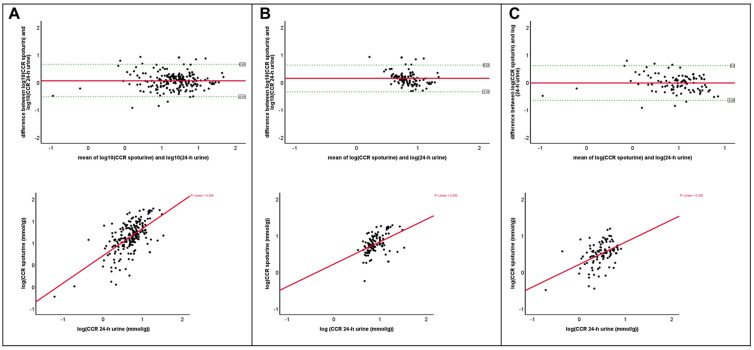
Bland–Altman plot and correlations of log-transformed spot urine CCR and log-transformed 24 h urine CCR in all patients (**A**), patients with hypercalciuria (**B**) and patients without hypercalciuria (**C**).

However, CCR values were significantly different between spot urine and 24 h urine (paired *t*-test, *p* < 0.001). To assess the agreement between the measurement methods, a Bland–Altman analysis was performed. The values were logarithmically transformed to achieve a normal distribution, which is required for conducting a Bland–Altman analysis. This ensured a more stable variance in the differences and a better approximation of the method’s assumptions. The results were subsequently back-transformed using exponentiation to represent the multiplicative range of agreement. The Bland–Altman plot of the differences in the CCR between spot and 24 h urine across all patients demonstrated moderate agreement between the methods (Bland–Altman bias = 1.06, LoA 0.59 to 1.91). A significant, yet also moderate, positive correlation was observed (Pearson’s r = 0.63, *p* < 0.001, ICC = 0.767, *p* < 0.001) (Figure 1A).

Bland–Altman analysis of patients with hypercalciuria showed a higher bias than for patients without hypercalciuria (1.15 vs. 0.97, independent sample *t*-test, *p*-value <0.001) whereas multiplicative limits of agreement were narrower (LoA 0.70 to 1.89 vs. 0.52 to 1.82, independent sample *t*-test *p*-value < 0.001) (Figure 1B,C). These subgroup findings indicate that the degree and direction of disagreement differed according to hypercalciuria status, but the limits of agreement remained too wide to support the interchangeability of spot urine and 24 h urine measurements. The sensitivity of the CCR with a cut-off value of >5 mmol/g in spot urine for detecting hypercalciuria was only 46.3%, whereas the specificity was 87.5%.

Although the calcium values were adjusted using the creatinine clearance ratio, impaired renal function could reduce the accuracy of spot urine measurements. We therefore performed a subgroup analysis and excluded patients with GFR values < 60 mL/min/1.73 m^2^ (n = 28). Additionally, creatinine values below 0.01 g/d/kgKG (n = 15) and 24 h urine samples with volumes < 500 mL (n = 2), which most likely indicate an error in urine sampling, were excluded, as well as values where either weight, GFR or urine volume was missing. However, Bland–Altman analysis showed almost the same bias of 1.05 (n = 137, LoA 0.61 to 1.83 mmol/g), with a notable decrease in the correlation between the measurement methods (Spearman’s r = 0.53, *p* < 0.001, Pearson’s r = 0.495, *p* < 0.001, ICC = 0.655, *p* < 0.001). Thus, exclusion of patients with indicators of impaired renal function or potentially incomplete collection did not materially improve agreement.

**Figure 2:**
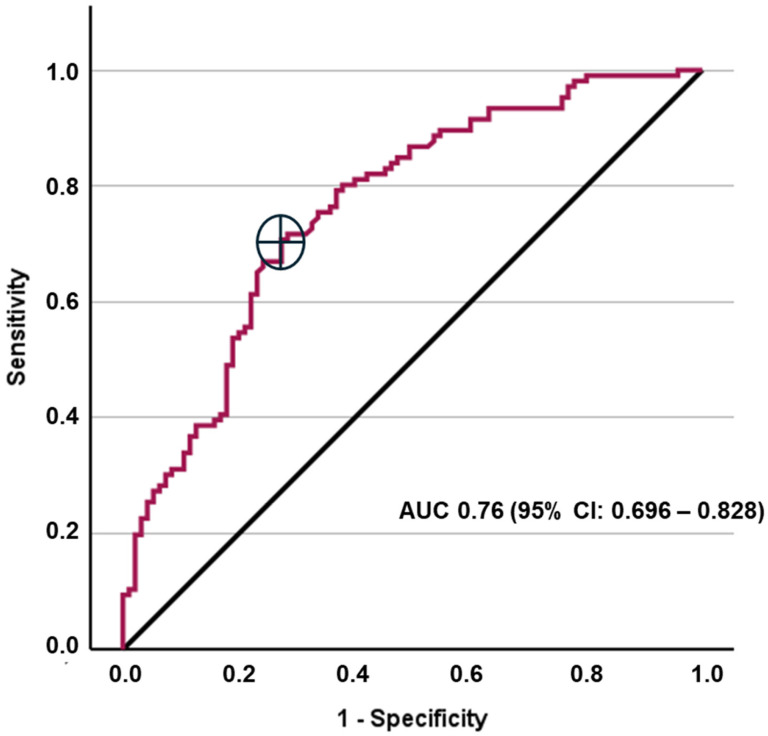
Receiver operating characteristic curve for spot urine CCR to predict hypercalciuria defined by 24 h urinary calcium excretion. The ROC-derived optimal cut-off was 4.4 mmol/g.

A ROC analysis was performed to evaluate the diagnostic performance of CCR of spot urine compared to the gold standard of 24 h urinary calcium excretion. The area under the ROC curve (AUC) was 0.76 (*p* = 0.03), indicating a moderate discriminative ability. The optimal cut-off value for spot urine calcium to predict hypercalciuria was determined to be 4.4 mmol/g, with a sensitivity of 70.8% and a specificity of 72.4% (Figure 2). The positive predictive value (PPV) and negative predictive value (NPV) were 0.74 and 0.69, respectively.

In a separate exploratory rule-out analysis, a lower spot urine CCR cut-off of 2 mmol/g showed a high sensitivity of 95.3% but a low specificity of 24.2%, with a positive predictive value of 0.42 and a negative predictive value of 0.82. Only 23 patients (11.4%) had a spot urine CCR below 2 mmol/g. Therefore, this low threshold may be useful only in a small subgroup and should not be interpreted as a general diagnostic cut-off.

In addition to impaired renal function, calcium excretion is notably influenced by multiple factors, including dietary calcium intake, and medication, all of which may contribute to discrepancies between spot urine and 24 h urine measurements. To evaluate the impact of potential confounding variables on the observed differences between these measurements, we performed a linear regression analysis. Using stepwise backward elimination, primary hyperparathyroidism and serum calcium remained statistically significant in the final model. Primary hyperparathyroidism was associated with greater disagreement between methods (exponentiated regression coefficient 1.212, *p* < 0.001), whereas higher serum calcium was associated with a lower spot-to-24 h CCR ratio (exponentiated regression coefficient 0.784, *p* = 0.01). Chronic kidney disease, osteoporosis, hypoparathyroidism, calcium intake, and cinacalcet therapy were retained in the exploratory model but did not reach conventional statistical significance (*p* = 0.05 to 0.09) and should therefore be interpreted as hypothesis-generating only (Table 2).

## 4. Discussion

The 24 h urine collection method involves the collection of all urine over a 24 h period, ensuring that any fluctuations in urinary calcium are captured accurately. If performed correctly, this method is highly sensitive and allows for the precise calculation of calcium excretion. In certain conditions, such as primary hyperparathyroidism, hypoparathyroidism, nephrolithiasis, and rare disorders like familial hypocalciuric hypercalcemia, the precise measurement of urinary calcium is essential for determining the appropriate therapeutic approach [20]. Nevertheless, the collection of urine over a 24 h period is associated with a significantly high error rate. Our study demonstrates that 16 patients (8%) exhibited very low creatinine levels (<10 mg/d/kgKG) in their urine or reduced collection volumes (<500 mL/d: n = 2, 1%), which could have served as indicators of inadequate collection quality. A retrospective analysis of 193 patients treated for kidney and/or ureteral stones between August 2014 and May 2016 showed that 42.5% of patients submitted the required 24 h urine sample. Of these, 34.1% were inadequate due to creatinine levels [21]. Another study examined the impact of an intervention to improve compliance with 24 h urine collection. After the intervention, compliance increased from 46.9% to 65.1% (*p* < 0.001). Adequate insurance coverage was associated with increased compliance (58.3% vs. 37.15%; *p* = 0.017) [22]. A limitation of our study is that the quality of the 24 h urine samples could not be adequately assessed, as the collection was not performed under controlled conditions. Deviations between spot urine and 24 h urine measurements may therefore partly reflect the limitations of the reference method, particularly if 24 h urine collection was incomplete or inaccurate.

The limitations of 24 h urine collection and the potential alternative of measuring CCR in spot urine have already been explored and investigated, particularly in children. Itami et al. analyzed the correlation between spot oxalate:creatinine ratio and 24 h oxalate excretion in the urine of children with and without primary hyperoxaluria, reporting a high correlation (r = 0.966, *p* < 0.001) [23]. A study from 2020 with 101 children confirmed the high correlation with calcium excretion [24]. Conversely, in adults the results could not be reproduced. Hashmi et al. found a poor correlation between those parameters in adult patients with kidney stones (r = 0.29, *p* = 0.005) [25], and Ilich et al. reported slightly better but still insufficient results in postmenopausal women (r = 0.6, *p* < 0.001) [26]. Our results are similar to those of the latter study.

The present study adds to the previous literature by evaluating spot urine CCR in a heterogeneous adult cohort from multiple centers, including patients from endocrine, nephrological, and endocrine surgical settings. This real-world design is clinically relevant because urinary calcium is frequently assessed in patients with complex disorders of mineral metabolism rather than in narrowly selected populations. However, this heterogeneity also limits the generalizability of the findings to any single diagnostic subgroup.

To minimize potential errors due to improper urine collection, patients with indicators of incomplete or incorrect collection were excluded. However, we did not observe any improvement in the correlation and therefore concluded that this was not a primary factor influencing the results. Some studies suggest that nutritional intake influences calcium levels in spot urine and have compared fasting and postprandial spot urine samples. However, neither approach demonstrated superior accuracy in detecting hypercalciuria [10]. Interestingly, our regression analysis identified an association between calcium intake and discrepancies in test results. Circadian calcium excretion could also be a reason for the inconsistent results. Although such an influence is well-established for potassium excretion, its impact on calcium excretion has not been thoroughly investigated [27]. Nevertheless, our study reflects real-world clinical practice, where precise control based on circadian rhythm or food intake cannot be reliably implemented.

Compared to previous studies, our study includes a larger and more heterogeneous patient cohort, with a significant proportion exhibiting hypercalciuria. Additionally, the number of patients with reduced renal function was relatively high, allowing us to demonstrate that this potential confounding factor could be mitigated by correcting for the calcium/creatinine ratio. Notably, this adjustment did not affect the overall agreement between the tests. The regression analysis suggested that disagreement was more pronounced in patients with primary hyperparathyroidism and varied with serum calcium. Other retained variables, including chronic kidney disease, osteoporosis, hypoparathyroidism, calcium intake, and cinacalcet therapy, did not reach statistical significance and should be interpreted cautiously. Importantly, in several of these clinical contexts, particularly parathyroid disorders and osteoporosis, accurate assessment of urinary calcium excretion is clinically relevant. Our findings therefore argue against replacing 24 h urine collection with spot urine CCR in these groups.

The results of our ROC analysis further confirm that spot urine cannot fully replace the measurement of calcium in 24 h urine. The ROC-derived optimal cut-off of 4.4 mmol/g showed only moderate sensitivity and specificity. A different clinical use may be a limited rule-out strategy: a spot urine CCR below 2 mmol/g showed high sensitivity and a negative predictive value of 82%. However, this low cut-off applied to only 23 patients, representing 11.4% of the cohort. Therefore, any potential reduction in 24 h urine collection would be limited to a small minority of patients. Spot urine CCR should not be interpreted as a broadly applicable replacement test or as sufficient for routine monitoring or follow-up in patients in whom accurate urinary calcium quantification is clinically important.

This study has several limitations. First, the retrospective design limited control over sampling conditions and introduced potential selection bias. Second, the cohort was heterogeneous, including patients with different underlying disorders and treatments that may have influenced urinary calcium excretion. Third, spot urine samples were random samples and were not standardized by time of day, fasting status, diet, or fluid intake. Fourth, the completeness of 24-h urine collection could only be assessed indirectly using urine volume and urinary creatinine excretion, and collection was not performed under controlled conditions.

Prospective studies with standardized sampling protocols are required to confirm whether very low spot urine CCR can safely be used as a rule-out threshold.

## 5. Conclusions

Spot urine CCR showed only moderate agreement and moderate diagnostic accuracy compared with 24 h urinary calcium excretion. Therefore, spot urine CCR cannot replace 24 h urine collection when an accurate assessment of urinary calcium excretion is required, particularly in patients with parathyroid disorders, nephrolithiasis, osteoporosis, renal impairment, or complex disturbances of calcium metabolism. A very low spot urine CCR below 2 mmol/g may help rule out hypercalciuria in a small subgroup of patients and could support triage decisions in selected cases. However, this potential application is limited by the small proportion of eligible patients and requires confirmation in prospective studies using standardized sampling protocols.

## Figures and Tables

**Table 1:** Baseline characteristics. Categorical variables are presented as n (%). Continuous variables are presented as median (IQR).

Patient Characteristics	Total, n (%)	Median	Q25	Q75
sex female	158 (78.6)			
age (years)		62	52	70
weight (kg)		71	60	84
height (cm)		168	160	175
BMI (kg/m^2^)		25.04	22.3	28.8
pre-existing conditions				
kidney				
stones	29 (14.4)			
nephrocalcinosis	1 (0.5)			
chronic kidney disease	30 (14.9)			
bone				
osteopenia	30 (14.9)			
osteoporosis	60 (29.9)			
DXA	107 (53.2)	−1.6	−2.4	−1
pre-existing fractures	13 (6.5)			
parathyroid				
primary hyperparathyroidism	133 (66.2)			
tertiary hyperparathyroidism	6 (3.0)			
hypoparathyroidism	17 (8.5)			
idiopathic hypercalciuria	4 (2.0)			
FHH	1 (0.5)			
medication				
vitamin D derivatives	108 (53.7)			
calcium	11 (5.5)			
natpar	2 (1)			
Cinacalcet	14 (7)			
bisphosphonates	13 (7.1)			
denosumab	1 (0.5)			
diuretics				
loop diuretics	19 (9.5)			
thiazide diuretics	10 (5.0)			
estrogens	2 (1.0)			
laboratory results				
calcium (mmol/L)	201 (100)	2.6	2.4	2.7
phosphate (mmol/L	198 (98.5)	0.99	0.81	1.2
albumin (g/dL)	201 (100)	4.4	4.1	4.7
PTH (pmol/L)	201 (100)	9.01	4.9	14
alkaline phosphatase (U/L)	193 (96.0)	80	60	98
25-OH vitamin D (nmol/L)	198 (98.5)	58	34	85
creatinine (mg/dL)	201 (100)	0.81	0.67	0.97
GFR (ml/min/1.73 m^2^)	180 (89.6)	79	63	96
calcium in spot urine (mmol/L)	201 (100)	3.2	1.9	4.9
creatinine in spot urine (g/L)	201 (100)	0.61	0.32	0.95
urine in 24 h (mL)	201 (100)	2200	1600	2800
calcium in 24 h urine (mmol/d)	201 (100)	5.7	3.2	9.2
creatinine in 24 h urine (g/d)	201 (100)	1.06	0.82	1.3
CCCR (%) spot urine	201 (100)	1.51	0.89	2.41
CCCR (%) 24 h urine	201 (100)	1.8	1.16	2.57
CCR (mmol/g) spot urine	201 (100)	4.42	3.13	6.96
CCR (mmol/g) 24 h urine	201 (100)	5.5	3.25	7.59

**Table 2:** Exploratory linear regression analysis of factors associated with disagreement between spot urine CCR and 24 h urine CCR after backward stepwise elimination. The dependent variable was the logarithmically transformed spot urine CCR/24 h urine CCR ratio. Regression coefficients are presented after exponentiation and therefore indicate multiplicative effects. Variables with *p* > 0.05 are interpreted as hypothesis-generating only.

Condition	exp. (Regression Coefficient)	*p*-Value	95% Confidence Interval	
constant	0.959	0.29	0.861	1.046
chronic kidney disease	1.115	0.09	0.984	1.265
osteoporosis	0.921	0.07	0.843	1.007
primary hyperparathyroidism	1.212	<0.001	1.09	1.348
hypoparathyroidism	0.795	0.06	0.623	1.014
calcium intake	1.289	0.09	0.963	1.724
cinacalcet	0.844	0.05	0.712	1.001
calcium (mmol/L)	0.784	0.01	0.649	0.947

## Data Availability

The data presented in this study are available on request from the corresponding author due to privacy reasons.

## Abbreviations

The following abbreviations are used in this manuscript:

AUC	Area Under the Curve
BÄK	German Medical Association (Bundesärztekammer)
CCR	Calcium-to-Creatinine Ratio
CCCR	Calcium–Creatinine Clearance Rate
DAkkS	German Accreditation Body (Deutsche Akkreditierungsstelle)
DGKL	German Society for Clinical Chemistry and Laboratory Medicine
FHH	Familial Hypocalciuric Hypercalcemia
GFR	Glomerular Filtration Rate
ICC	Intraclass Correlation Coefficient
ISO	International Organization for Standardization
LoA	Limits of Agreement
NPV	Negative Predictive Value
PPV	Positive Predictive Value
PTH	Parathyroid Hormone
ROC	Receiver Operating Characteristic
SD	Standard Deviation

## References

[1] Kanis J.A., Cooper C., Rizzoli R., Reginster J.Y. (2018). European guidance for the diagnosis and management of osteoporosis in postmenopausal women. Osteoporos. Int..

[2] Bollerslev J., Rejnmark L., Zahn A., Heck A., Appelman-Dijkstra N.M., Cardoso L., Hannan F.M., Cetani F., Sikjaer T., Formenti A.M. (2021). European expert consensus on practical management of specific aspects of parathyroid disorders in adults and in pregnancy: Recommendations of the ESE Educational Program of Parathyroid Disorders (PARAT 2021). Eur. J. Endocrinol..

[3] Skolarikos A., Geraghty R., Somani B., Tailly T., Jung H., Neisius A., Petřík A., Kamphuis G.M., Davis N., Bezuidenhout C. (2025). European Association of Urology Guidelines on the Diagnosis and Treatment of Urolithiasis. Eur. Urol..

[4] Worcester E.M., Coe F.L. (2008). New Insights Into the Pathogenesis of Idiopathic Hypercalciuria. Semin. Nephrol..

[5] Bilezikian J.P., Khan A.A., Silverberg S.J., Fuleihan G.E.-H., Marcocci C., Minisola S., Perrier N., Sitges-Serra A., Thakker R.V., Guyatt G. (2022). Evaluation and Management of Primary Hyperparathyroidism: Summary Statement and Guidelines from the Fifth International Workshop. J. Bone Miner. Res..

[6] Bollerslev J., Rejnmark L., Marcocci C., Shoback D.M., Sitges-Serra A., Van Biesen W., Dekkers O.M., European Society of Endocrinology (2015). European Society of Endocrinology Clinical Guideline: Treatment of chronic hypoparathyroidism in adults. Eur. J. Endocrinol..

[7] Alexander R.T., Dimke H. (2023). Effects of parathyroid hormone on renal tubular calcium and phosphate handling. Acta Physiol..

[8] Blaine J., Chonchol M., Levi M. (2014). Renal Control of Calcium, Phosphate, and Magnesium Homeostasis. Clin. J. Am. Soc. Nephrol..

[9] Smith L.M., Gallagher J.C. (2020). Reference range for 24-h urine calcium, calcium/creatinine ratio, and correlations with calcium absorption and serum vitamin D metabolites in normal women. Osteoporos. Int..

[10] Jones A.N., Shafer M.M., Keuler N.S., Crone E.M., Hansen K.E. (2012). Fasting and postprandial spot urine calcium-to-creatinine ratios do not detect hypercalciuria. Osteoporos. Int..

[11] Nouvenne A., Meschi T., Prati B., Guerra A., Allegri F., Vezzoli G., Soldati L., Gambaro G., Maggiore U., Borghi L. (2010). Effects of a low-salt diet on idiopathic hypercalciuria in calcium-oxalate stone formers: A 3-mo randomized controlled trial. Am. J. Clin. Nutr..

[12] Yü T.F., Berger L., Sarkozi L., Kaung C. (1981). Effects of Diuretics on Urate and Calcium Excretion. Arch. Intern. Med..

[13] Nordin B.E.C. (1959). Assessment of Calcium Excretion from the Urinary Calcium/Creatinine Ratio. Lancet.

[14] Gökçe Ç., Gökçe O., Baydinç C., Ilhan N., Alaşehirli E., Ozküçük F., Taşçi M., Atilkeler M.K., Celebi H., Arslan N. (1991). Use of Random Urine Samples to Estimate Total Urinary Calcium and Phosphate Excretion. Arch. Intern. Med..

[15] Christensen S.E., Nissen P.H., Vestergaard P., Heickendorff L., Brixen K., Mosekilde L. (2008). Discriminative power of three indices of renal calcium excretion for the distinction between familial hypocalciuric hypercalcaemia and primary hyperparathyroidism: A follow-up study on methods. Clin. Endocrinol..

[16] Xiang A., Nourian A., Ghiraldi E., Friedlander J.I. (2021). Improving Compliance with 24-H Urine Collections: Understanding Inadequacies in the Collection Process and Risk Factors for Poor Compliance. Curr. Urol. Rep..

[17] Mann S.J., Gerber L.M. (2019). Addressing the problem of inaccuracy of measured 24-h urine collections due to incomplete collection. J. Clin. Hypertens..

[18] Arrabal-Polo M.A., Arias-Santiago S., Girón-Prieto M.S., Abad-Menor F., Pintado F.L.-C., Zuluaga-Gomez A., Arrabal-Martin M. (2012). Hypercalciuria, hyperoxaluria, and hypocitraturia screening from random urine samples in patients with calcium lithiasis. Urol. Res..

[19] (2024). Medizinische Laboratorien—Anforderungen an die Qualität und Kompetenz.

[20] Weber T., Dotzenrath C., Dralle H., Niederle B., Riss P., Holzer K., Kußmann J., Trupka A., Negele T., Kaderli R. (2021). Management of primary and renal hyperparathyroidism: Guidelines from the German Association of Endocrine Surgeons (CAEK). Langenbeck’s Arch. Surg..

[21] Ghiraldi E.M., Reddy M., Li T., Lawler A.C., Friedlander J.I. (2017). Factors Associated with Compliance in Submitting 24-Hour Urine Collections in an Underserved Community. J. Endourol..

[22] Boyd C., Wood K., Ashorobi O., Harvey L., Oster R., Holmes R.P., Assimos D.G. (2019). An Intervention to Increase 24-Hour Urine Collection Compliance. Urol. Pract..

[23] Itami N., Yasoshima K., Akutsu Y., Nonomura K. (1990). Spot-urine screening for primary hyperoxaluria. Nephron.

[24] Paccaud Y., Rios-Leyvraz M., Bochud M., Tabin R., Genin B., Russo M., Rossier M.F., Bovet P., Chiolero A., Parvex P. (2020). Spot urine samples to estimate 24-h urinary calcium excretion in school-age children. Eur. J. Pediatr..

[25] Hashmi S.B., Jafri L., Majid H., Talati J., Aziz W., Khan A.H. (2020). Relationship of spot urine oxalate to creatinine ratio and 24 h urinary oxalate excretion in patients with urolithiasis. Ann. Med. Surg..

[26] Ilich J.Z., Blanuša M., Orlić Ž.C., Orct T., Kostial K. (2009). Comparison of calcium, magnesium, sodium, potassium, zinc, and creatinine concentration in 24-h and spot urine samples in women. Clin. Chem. Lab. Med..

[27] Costello H.M., Johnston J.G., Juffre A., Ryan Crislip G., Gumz M.L. (2022). Circadian clocks of the kidney: Function, mechanism, and regulation. Physiol. Rev..

